# Central Composite Design for Formulation and Optimization of Solid Lipid Nanoparticles to Enhance Oral Bioavailability of Acyclovir

**DOI:** 10.3390/molecules26185432

**Published:** 2021-09-07

**Authors:** Haniza Hassan, Siti Khadijah Adam, Ekram Alias, Meor Mohd Redzuan Meor Mohd Affandi, Ahmad Fuad Shamsuddin, Rusliza Basir

**Affiliations:** 1 Department of Human Anatomy, Faculty of Medicine and Health Sciences, University Putra Malaysia (UPM), Serdang 43400, Malaysia; sk.adam@upm.edu.my (S.K.A.); rusliza@upm.edu.my (R.B.); 2 UKM Medical Centre, Department of Biochemistry, Faculty of Medicine, Universiti Kebangsaan Malaysia, Jalan Yaakob Latiff, Bandar Tun Razak, Kuala Lumpur 56000, Malaysia; ekram.alias@ppukm.ukm.edu.my; 3 School of Pharmacy, Puncak Alam Campus, Universiti Teknologi MARA (UiTM), Bandar Puncak Alam, Shah Alam 42300, Malaysia; meor@uitm.edu.my; 4 Faculty of Pharmacy and Health Sciences, Universiti Kuala Lumpur Royal College of Medicine Perak, Ipoh 30450, Malaysia; fuad.shamsuddin@unikl.edu.my

**Keywords:** central composite design, solid lipid nanoparticles, acyclovir, bioavailability, oral delivery

## Abstract

Treatment of herpes simplex infection requires high and frequent doses of oral acyclovir to attain its maximum therapeutic effect. The current therapeutic regimen of acyclovir is known to cause unwarranted dose-related adverse effects, including acute kidney injury. For this reason, a suitable delivery system for acyclovir was developed to improve the pharmacokinetic limitations and ultimately administer the drug at a lower dose and/or less frequently. In this study, solid lipid nanoparticles were designed to improve the oral bioavailability of acyclovir. The central composite design was applied to investigate the influence of the materials on the physicochemical properties of the solid lipid nanoparticles, and the optimized formulation was further characterized. Solid lipid nanoparticles formulated from Compritol 888 ATO resulted in a particle size of 108.67 ± 1.03 nm with an entrapment efficiency of 91.05 ± 0.75%. The analyses showed that the optimum combination of surfactant and solid lipid produced solid lipid nanoparticles of good quality with controlled release property and was stable at refrigerated and room temperature for at least 3 months. A five-fold increase in oral bioavailability of acyclovir-loaded solid lipid nanoparticles was observed in rats compared to commercial acyclovir suspension. This study has presented promising results that solid lipid nanoparticles could potentially be used as an oral drug delivery vehicle for acyclovir due to their excellent properties.

## 1. Introduction

Acyclovir (C_8_H_11_N_5_O_3_) is an antiviral drug derived from a guanosine analog. It is the gold standard drug prescribed by physicians as first-line therapy and prophylactic treatment for herpes simplex virus (HSV) infections. Acyclovir is marketed in several forms, including oral tablets and suspensions, topical ointment, and intravenous infusion. During viral outbreaks, treatment with acyclovir helps to accelerate the time to recovery by interfering with the replication process through competitive inhibition of viral DNA formation [1,2].

In the last decade, acyclovir has gained worldwide attention, especially among pharmaceutical researchers and health care practitioners. Classified as a class III drug in the Biopharmaceutics Classification System (BCS), orally administered acyclovir accounts for only 20–30% of the total free drug in the systemic circulation due to its poor water solubility [3,4]. The half-life of the drug is short; up to 2.5 h. Therefore, frequent and high doses of oral administrations of acyclovir (200 mg, 5 times daily) are required to maintain the therapeutic effect, depending on the pharmacokinetic profile of immunocompetent patients [5]. Nevertheless, the current therapeutic regimen is not patient compliant and adverse effects, such as acute renal impairment, are to be expected. An innovative and advanced approach to improve the pharmacokinetic limitations and further reduce the side effects of acyclovir, such as a novel drug delivery system [6] or supramolecular strategies, is desired [7].

In the past, various pharmaceutical formulations, such as lipid-based nanoparticles [8], polymeric nanoparticulate systems [9], mixed micelles and niosomal carriers [10,11] have been studied as strategies to increase the solubility and bioavailability of poorly soluble drugs. Several attempts have also been made to incorporate acyclovir into different types of delivery systems, including microemulsion [12], liposome [13] and nanoemulsion [14] to enhance its bioavailability [6,15]. However, the emulsion system was not very stable during storage, with drug leakage and particle size growth observed due to the coalescence of nanoparticles [16]. In the last decade, solid lipid nanoparticles (SLN), which offer many advantages over conventional colloidal drug carriers, have been proposed as a potential carrier vehicle [17]. It is postulated that this system could be an alternative delivery system due to its biocompatibility, biodegradability and stability and most importantly, it can overcome the pharmacokinetic limitations faced by newly synthesized and commercially available drugs. A study reported that acyclovir was successfully encapsulated into SLN with good physicochemical properties and morphology, including good encapsulation efficiency for ocular delivery of acyclovir [18].

Apart from this, formulations of SLN for oral drug delivery provide surface protection against biodegradation of the encapsulated drug and improve the uptake of the active compounds, as SLN can penetrate the epithelial cells [19,20]. A study also reported that cyclosporine A loaded in SLN had better oral bioavailability compared to cyclosporine A nanocrystals under the same experimental conditions [21]. Studies also found that cyclosporine A loaded in SLN dispersion exhibited sustained-release property and improved in its solubility [22,23]. These results highlight the potential of SLN for oral drug delivery over other carrier systems. 

Once a proposed nanocarrier system is identified, one of the crucial steps during its development is the design and optimization. Various techniques and approaches can be used for the design of nanoparticle formulation. The conventional approach, which is most commonly practiced, is to change a single factor or variable while keeping the other independent factors constant to observe the effect of composition or process variables on quality attributes. However, this approach requires a large number of experiments, and the interaction between factors is difficult to study. The results of the experiments could also be misinterpreted [24]. To overcome this problem, the application of a design of experiment methods, such as Central Composite Design (CCD) of Response Surface Methodology (RSM), during the design and development process could simultaneously determine the interactive effect of different variables that influence the results/quality of products (responses). Moreover, CCD has been successfully used in several studies for the development and optimization of formulations, as the data obtained with CCD showed good and reliable predictions [25,26]. 

Therefore, it is an innovative idea that, in this study, CCD was employed to optimize the SLN formulation for encapsulation of acyclovir by investigating the effect of two independent variables, the composition of Compritol 888 ATO (solid lipid) and Tween 80 (surfactant), on three dependent variables, namely particle size, polydispersity index (PdI) and zeta potential. The application of CCD in this study was also expected to save experimental time, human and natural resources. The optimized formulation of acyclovir-loaded SLN was also evaluated for its pharmacokinetic profile to support the study hypothesis; acyclovir-loaded SLN increases the oral bioavailability and absorption of acyclovir when administered orally in an in vivo model.

## 2. Results

### 2.1. Fitting the Response Surface Model

The variation in size, zeta potential and polydispersity index (PdI) of SLN were predicted using response surface methodology as these responses depend on the composition of nanoparticles. All data were statistically analyzed and used to determine the best-fit model for the independent variables of SLN. The regression coefficients (R^2^), regression value (*p*-value) and derived equations for particle size, polydispersity index and zeta potential are shown in Table 1. The non-significant linear terms (*p* > 0.05) were also included in the final reduced model if the quadratic or interaction terms containing these variables were significant (*p <* 0.05).

**Figure 1:**
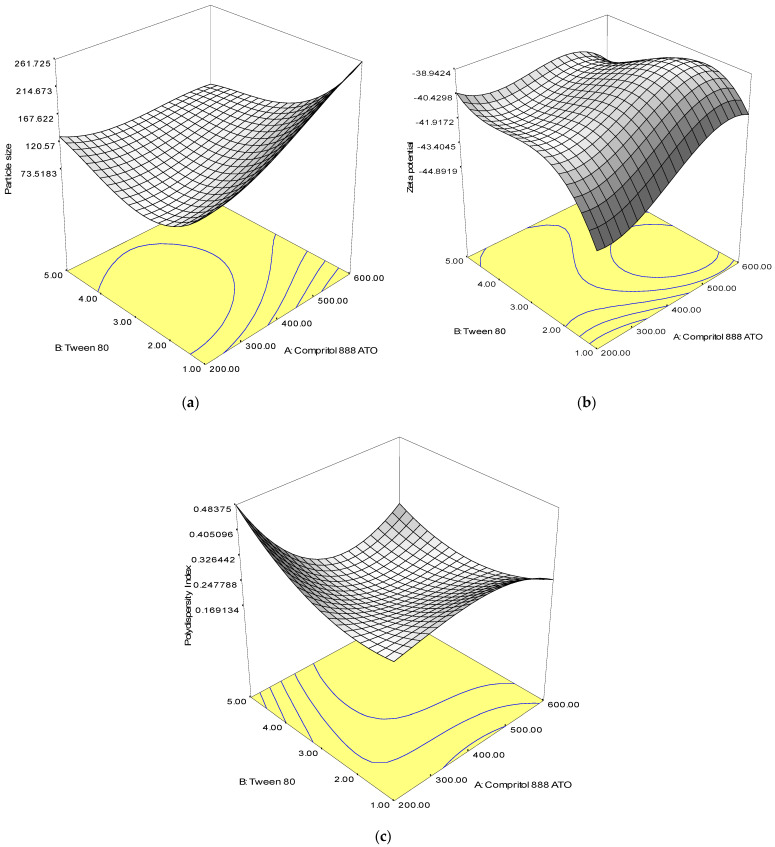
Response surface plots showing the effect of interaction between Compritol 888 ATO and Tween 80 composition on the (**a**) particle size, (**b**) zeta potential and (**c**) polydispersity index.

Analysis of variance (ANOVA) was used to assess the significance of the quadratic polynomial models developed (Table 2). The large F-value and small *p*-value (*p* < 0.05) of all terms in the models indicated significant influence on the response variables. From the result, the solid lipid (Compritol 888 ATO) had a positive effect on the size and zeta potential, while the concentration of surfactant (Tween 80) had a significant effect on the PdI value of SLN. The interaction between solid lipid and surfactant showed a significant effect on the particle size, zeta potential and polydispersity index of SLN. The 3D response surface plots of the combination of solid lipid and surfactant further explained the interaction between the factors in the design (Figure 1).

An optimal combination of solid lipid and surfactant is critical, as an increase in average particle size, zeta potential, and polydispersity index was observed when the concentration of lipid and surfactant was increased. Formulations with smaller particle size, lower polydispersity index and zeta potential can be prepared with a low concentration of surfactant.

### 2.2. Verification of Reduced Model 

From the observations, there were no significant differences (*p* < 0.05) between the experimental and predicted values for all responses by the response surface model derived from the proposed final composition of the solid lipid and surfactant (Table 3).

### 2.3. Physical Characteristics and Entrapment Efficiency of Acyclovir-Loaded SLN

The percentage of drug entrapment for acyclovir-loaded SLN was high, 91.05 ± 0.75%. The average particle size, PdI value, and zeta potential of acyclovir-loaded SLN measured by a dynamic light scattering (DLS) method were 108.67 ± 1.03 nm, 0.22 ± 0.03, and −33.45 ± 0.78, respectively. Data suggested that incorporation of acyclovir did not significantly affect the physical properties of the SLN system.

### 2.4. Transmission Electron Microscopy

**Figure 2:**
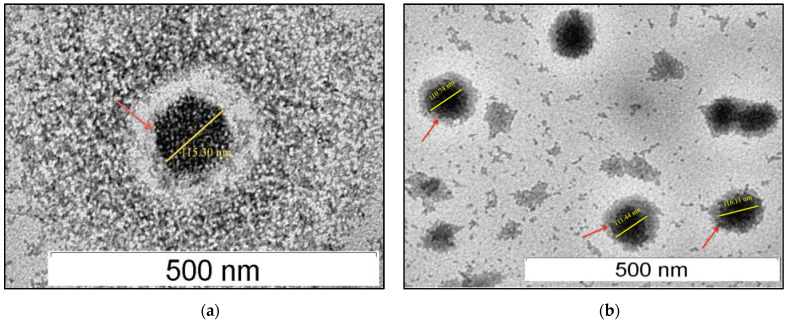
Transmission electron micrograph of drug-free SLN (**a**) and acyclovir-loaded SLN (**b**) at 60,000× magnification.

Observation under a transmission electron microscope (TEM) revealed that the average size of the blank and SLN loaded with acyclovir was less than 150 nm (Figure 2), which was consistent with the data from DLS. All samples showed a well-dispersed and homogeneous particle distribution with the typical spherical shape of SLN.

### 2.5. Differential Scanning Calorimetry Analysis

Acyclovir is known to have a melting temperature of 255.0 °C at 99% purity (Sigma Aldrich clinical datasheet). Prior to SLN preparation and optimization, a confirmatory test was performed, and the pure acyclovir compound showed a single peak at 249.3 °C, which is close to the manufacturer’s melting temperature specifications. Therefore, the method of hot homogenization with ultrasonication chosen for the preparation of acyclovir-loaded SLN in this study was suitable, as acyclovir remained in its crystal form at a high temperature of 75.0 °C.

**Figure 3:**
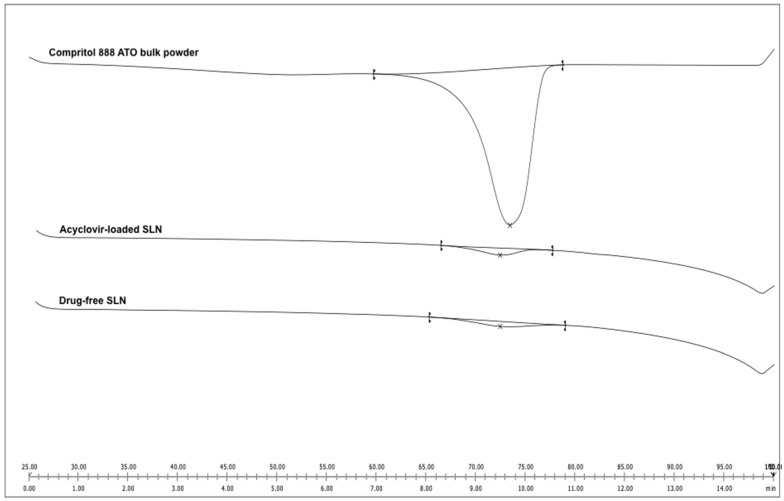
Differential scanning calorimetry thermograms of Compritol 888 ATO bulk powder, acyclovir-loaded SLN and drug-free SLN (blank).

From the thermogram (Figure 3), slight temperature shifts to 72.13 and 72.12 °C for the drug-free (blank) and acyclovir-loaded SLN, respectively (the melting temperature of bulk Compritol 888 ATO was 72.74 °C), indicating an interaction between lipid and surfactant. The melting enthalpy of bulk Compritol 888 ATO was 132.85 J/g and decreased to 4.63 and 4.53 J/g for the empty and acyclovir-loaded SLN, respectively. The recrystallization index (RI) was 87.12% and 85.24% for the blank and acyclovir-loaded SLN, respectively, while the bulk solid lipid was set at 100%.

### 2.6. Short-Term Stability Test

The size, PdI and zeta potential of the drug-free and acyclovir-loaded SLN dispersions stored at refrigerated and room temperature showed no significant changes (*p* > 0.05) and remained stable after the day of manufacture for up to three months of storage, maintaining their original size, PdI and zeta potential (Table 4). However, when the drug-free and acyclovir-loaded SLN dispersions were stored at an extreme temperature of 40 °C, the mean particle size of both formulations increased dramatically after three months of storage (*p* < 0.001). The PdI values of both SLN dispersions observed after three months were also high with subsequent formation of gel (gelation). A significant decrease (*p* < 0.001) in zeta potential value of both SLN dispersions was also noticed when compared with the corresponding freshly prepared SLN dispersions.

### 2.7. In Vitro Release Study

**Figure 4:**
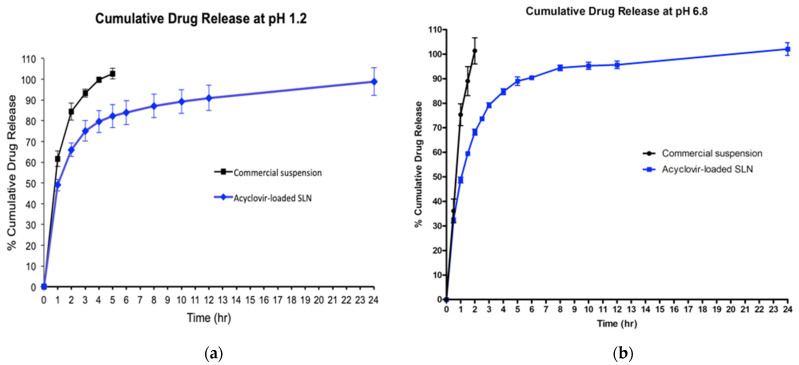
Cumulative percentages of acyclovir release profiles from acyclovir-loaded SLN and commercial oral suspension at pH of 1.2 (**a**) and pH of 6.8 (**b**).

The in vitro release study showed that the commercial acyclovir suspension achieved a cumulative release of 100% in five hours when tested at a pH of 1.2, while a shorter release time of about 2 h was observed at a pH of 6.8. In contrast, the release profile of acyclovir from SLN dispersion in both simulated gastric fluid (GIF) and simulated intestinal fluid (SIF) showed a biphasic release pattern with burst release during the initial state. Approximately 49% of the drug was released in the first hour, followed by sustained-release for up to 24 h (Figure 4). The two acyclovir-loaded SLN plots for pH 1.2 and 6.8 were almost superimposed.

### 2.8. In Vivo Pharmacokinetic Evaluation

**Figure 5:**
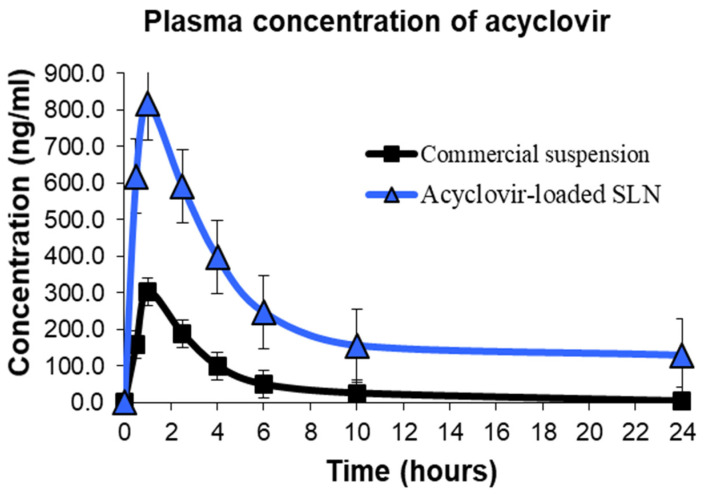
Plasma concentration versus time profile after oral administration of acyclovir-loaded SLN or commercial suspension in rats.

Rats administered with acyclovir-loaded SLN showed the highest plasma concentration profile (C_max_) with a mean of 818.67 ng/mL, about 2.7 times higher than the mean maximum concentration of the commercial suspension group (Table 5). Statistical analysis showed that the rat administered with acyclovir-loaded SLN had significantly higher drug absorption compared to the commercial oral acyclovir suspension (*p* < 0.05). The time to reach maximum (T_max_) acyclovir plasma concentration was similar for all treatment groups and was reported in the first hour after oral administration (Figure 5).

The AUC calculated in the in vivo pharmacokinetics study represents the total amount of drug exposed over time and is an indicator of the bioavailability of a drug. The mean values of AUC_0–24_ and AUC_0–∞_ for the commercial acyclovir suspension and acyclovir-loaded SLN are illustrated in Table 5. A significantly higher AUC_0–24_ and AUC_0–∞_ of acyclovir-loaded SLN was obtained (*p* < 0.05) when compared to the commercial suspension. From the AUC data, the relative bioavailability of acyclovir-loaded SLN was 505.57% (commercial acyclovir suspension was used as a reference). The elimination constant (K_e_) for commercial acyclovir suspension and acyclovir-loaded SLN was 0.37 and 0.15, respectively. The half-life (t_1/2_) of acyclovir-loaded SLN was 5.53 h, a significant increase compared with the mean half-life of acyclovir suspension of 2.06 h.

### 2.9. Histological Observation under the Light Microscope

**Figure 6:**
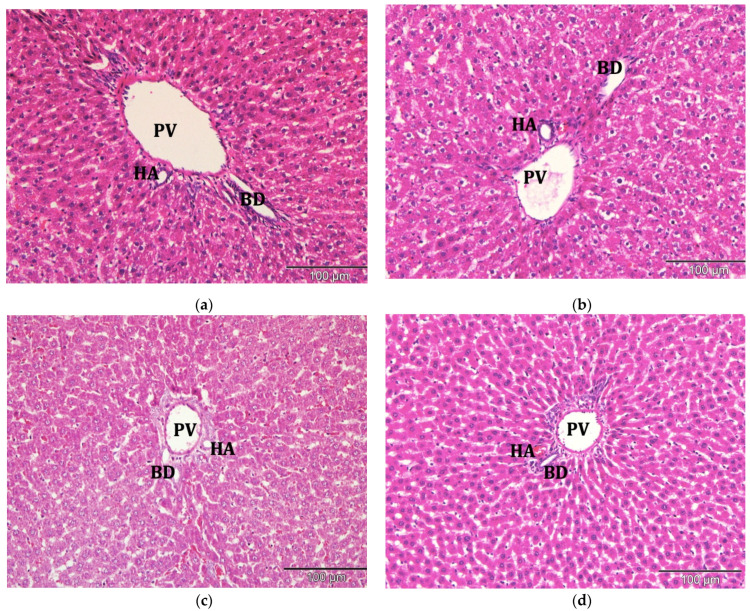
Photomicrograph of liver tissues from (**a**) control; (**b**) commercial acyclovir suspension; (**c**) drug-free SLN and (**d**) acyclovir-loaded SLN treated group (H&E stain, 200×). BD: bile duct, HA: hepatic artery, PV: portal vein.

Histological examination of liver sections from all treatment groups showed no significant structural abnormalities compared to control (Figure 6). Hematoxylin and eosin (H&E) staining of all liver tissues showed normal hepatocytes with intact cell membrane, which looked similar to the healthy liver when viewed under a light microscope. It could be concluded that no pathological change of liver tissue was detected in all groups.

**Figure 7:**
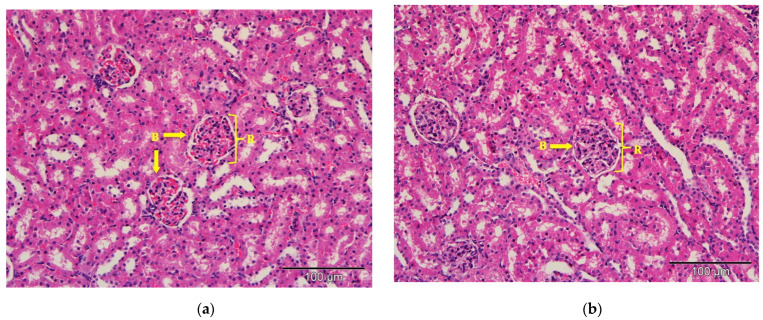

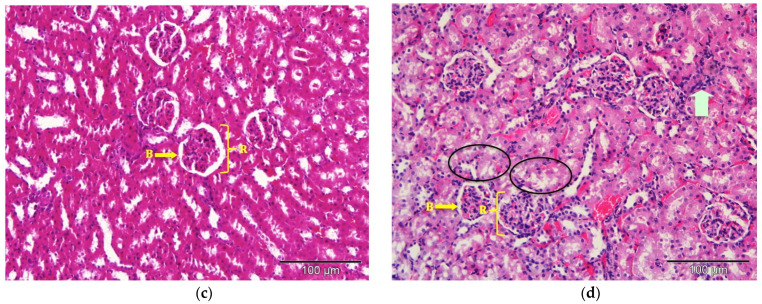
Photomicrograph of kidney tissues from (**a**) control; (**b**) commercial acyclovir suspension; (**c**) drug-free SLN and (**d**) acyclovir-loaded SLN treated groups (H&E stain, 200×). Interstitial infiltration of inflammatory cells (arrowed) and acute necrosis of renal tubules (circle). B: Bowman’s capsule, R: renal corpuscles.

The kidney tissues architecture of the rats treated with saline (control), commercial acyclovir suspension or drug-free (blank) SLN showed no pathological changes. The morphology of the tissues also appeared to be normal, as shown in Figure 7. The structure of glomeruli and tubules of the nephrons, as well as the blood vessels, remained unchanged. However, interstitial infiltration of inflammatory cells in the kidney was observed in the acyclovir-loaded SLN groups, as shown in Figure 7d. A few of the renal tubules had also become necrotic (acute). Despite this, no substantial lesions were found in the glomerular tissues.

## 3. Discussion

When designing and developing SLN formulations, CCD may be utilized to gain a better understanding of how the magnitude of a single factor and its interaction affect the responses. In this study, when high lipid content was introduced to the system, it resulted in the production of larger particles because the disruption process of a hot oil droplet was difficult, and the breakdown rate was retarded due to high viscosity and flow resistance [27,28]. Furthermore, particle aggregation could occur as a result of hydrophobic interaction between the particles if the surfactant molecules present were insufficient to cover the surface of the nanoparticles and the collision rate of SLN formed was faster than the surfactant molecules to adsorb onto the particles [29]. 

The finding of high PdI value of the formulation in this study could be explained by increased number of non-uniform micelles formation following the addition of excessive surfactant into the system [30]. The greater flow resistance caused by higher viscosity of the aqueous phase in the presence of a high concentration of surfactant might impact the emulsification efficiency during SLN preparation. Particles of varying sizes were formed due to an increase in the coalescence rate of lipid emulsion. A formulation with smaller particle sizes and lower PdI could be produced with an optimal concentration of solid lipid and surfactant during the fabrication process.

The increment in surfactant concentration had caused the molecules to accumulate and adsorp onto the surface of solid lipid nanoparticles, shielding the particles’ surface charge [31], and hence, dropped the zeta potential value of the SLN system [32]. Although the adsorption of a non-ionic surfactant may result in a decrease in zeta potential value, this is not the only indication of a decrease in electrostatic repulsion. All responses were affected by the interaction between the independent variables (AB), which represents a quadratic relationship.

Examination under a TEM found that the blank and acyclovir-loaded SLN developed had a typical spherical shape with a smooth surface, similar to other previous studies [28,30,33]. The data also indicated that SLN had relatively high entrapment efficiency, suggesting that acyclovir was adequately encapsulated in the lipid melt during its production [34]. In theory, when a small amount of drug is added to the SLN formulation, ample space in the lipid is available to accommodate the drug molecules [30]. The high percentage of acyclovir encapsulation efficiency observed in this study may be due to the solid lipid content of Compritol 888 ATO. This type of lipid is made up of a combination of monoglycerides, diglycerides, and triglycerides that can create an irregular or less ordered crystalline structure (lipid matrices), allowing for a larger proportion of drug entrapment [35]. 

The thermogram of the lipid in both blank and acyclovir-loaded SLN showed a lower melting temperature, onset temperature and melting enthalpy compared to their bulk lipid counterpart, indicating an interaction between the surfactant and solid lipid apart from having a higher surface area to volume ratio due to its small size (Figure 3) [36]. This finding could also be linked to the formation of lattice defects in lipid matrices, which leads to a decrease in lipid crystallinity in SLN. When compared to the highly crystalline bulk lipid, the less ordered lipid structure of SLN required less energy to break the lattice forces and melt the lipid [37,38]. The degree of crystallinity (RI) of SLN was calculated to support these findings, and data were consistent. A less ordered arrangement of the lipid crystalline structure is critical in the development of SLN as a drug delivery vehicle because it determines whether the drug molecules can be firmly encapsulated into the carrier or expelled from the system. SLN with a lower level of crystal lattice organization resulted in a higher percentage of entrapment efficiency and controlled drug release [39,40]. 

This study was also interested in assessing the release of acyclovir-loaded SLN in the simulated gastrointestinal environment using simulated gastric fluid (pH 1.2) and simulated intestinal fluid (pH 6.8) at body temperature (37 °C). The in vitro drug release study in both pH levels suggested that the release of acyclovir from SLN was not affected by different pH values where comparable drug release patterns were observed in pH 1.2 and pH 6.8 release media, similar to the result reported by another researcher [41]. Apart from that, the release of acyclovir from its delivery system showed a surface phenomenon, indicating the presence of acyclovir on the nanoparticle surface. This model is referred to as a drug-rich shell model. This type of SLN model is often produced, especially after the hot homogenization process. The high temperature employed during the initial synthesis of SLN increased the solubility of acyclovir, causing it to partition into the aqueous phase, and only a small amount of drug remained in the hot lipid melt. The drug molecules then re-partitioned into the lipid phase from the aqueous phase resulting in drug enrichment on the outer layer of the SLN [30,35]. Furthermore, the finding of this study also showed that the rate of acyclovir release from SLN was much slower than that of commercial acyclovir suspension, which suggested entrapment of a portion of the drug in the solid lipid core [42]. This result also indicated that the carrier system allows sustained/prolonged drug release. 

The physical stability test is required for the successful development of a formulation. To avoid the potential influence of light on the SLN stability test, SLN dispersions were kept in amber colored storage bottles in this study. At 4 and 25 °C, SLNs were considered stable. These findings could be attributed to the full coverage of the non-ionic surfactant, Tween 80, which causes steric hindrance, as well as electrostatic repulsion from the solid lipid [43]. Surprisingly, this was not the case for SLN that was developed using Biogapress Vegetal 297 ATO as solid lipid material, in our previous study [8]. Although the same methods and optimization processes were employed to develop this nanocarrier system, after the first month of storage, the size of the blank Biogapress Vegetal 297 ATO SLN and acyclovir-loaded Biogapress Vegetal 297 ATO SLN formulations stored at room temperature started to increase significantly, and high polydispersity index values were recorded. This is an indication of particle aggregation and instability of the system, apart from lower zeta potential measurement (unpublished data). In most cases, a lack of surfactant promotes particle collision and aggregation due to hydrophobic interactions between nanoparticles, which can be avoided during storage at low temperatures.

SLN was found to be unstable at 40 °C due to low microviscosity of the formulations. Microviscosity is a firm layer of emulsifier that restricts the fusion of film layers that exist on the SLN surface as a result of particle contact, and it is temperature-dependent [44]. The zeta potential measurements also corresponded well with the instability of SLN dispersions observed at this temperature. A stable SLN dispersion could be obtained if the electrostatic repulsion is greater than the attractive van de Waals forces since nanoparticles have to overcome the strong repulsive barrier to get closer to each other and form aggregates [35]. Another possible explanation for the decreased zeta potential measurements is that high temperature (high energy input) would cause structural changes in the solid lipid crystalline matrix. The re-orientation of the particle matrices (crystallinity) may affect particle surface charges, resulting in changes in the zeta potential measurement. High storage temperatures are also known to increase the kinetic energy of the SLN system, which was sufficient to overcome the electrostatic repulsion and promote particle collision [44,45]. 

Intestinal permeability and oral bioavailability of acyclovir are improved when loaded into SLN due to several mechanisms. An earlier study found that SLN could enter the lymphatic circulation via two mechanisms before draining into the thoracic duct and transferred to venous blood. The first mechanism involved nanoparticles uptake via microfold cells (M cells) in the Peyer’s patches of intestine, while the second involved paracellular and/or transcellular SLN uptake [46]. Therefore, drugs encapsulated in SLN can be transported as a particulate form to the lymphatic circulation by both mechanistic approaches. Administration of drug-loaded SLN via the oral route also may result in the formation and release of free drug molecules and chylomicron by enterocytes in the gut as a result of triglyceride (solid lipid) digestion, similar to digestion and absorption of long-chain fatty acid from food. By producing a chylomicron-drug mixture, this event would promote subsequent drug uptake into enterocytes. With regard to the aforementioned mechanistic approaches, further enhancement of intestinal permeation and drug absorption was anticipated [20]. The mechanistic approach of SLN uptake by intestinal cells was also found to be size-dependent; the smaller particle size, the higher chances of particle uptake by M cells of Peyer’s patches [47]. Smaller nanoparticles also had a better systemic uptake profile due to easier release from the Peyer’s patches, which aids in their transport into lymphatic circulation [48]. Nevertheless, further investigations are required to be performed to determine the exact mechanisms of acyclovir-loaded SLN intestinal uptake in the future.

Histopathological examination of liver and kidney tissues confirmed that drug-free SLN is non-toxic. Weyhers and colleagues reported similar findings in an earlier study, demonstrating that low doses of unloaded SLN dispersion made from Compritol 888, soy lecithin, and Tween 80 were well tolerated in mice [49]. On the other hand, high levels of acyclovir in the systemic circulation of rats administered acyclovir-loaded SLN caused substantial histological changes in the kidney tissues. According to a previous study, high concentrations of acyclovir in the systemic circulation after oral delivery caused interstitial infiltrations of inflammatory cells in the kidney [50]. Apart from that, inadequate hydration during acyclovir therapy and prolonged drug contact with renal tubules may also cause kidney damage [51,52,53]. It should be recommended for future studies that a lower dose of acyclovir-loaded SLN is sufficient for effective therapy. 

## 4. Materials and Methods

The experiment workflow of the study is overviewed in Figure S1.

### 4.1. Materials

Acycloguanosine (Acyclovir, 99% pure chemical) and polysorbate 80 (Tween 80) were provided by Sigma-Aldrich (St Louis, MO, USA). Gattefosse (Lyon France) presented Glyceryl dibehanate (Compritol 888 ATO) as a gift. The Milli-Q filtering system was used to deionize the water. All compounds used in the study were of the highest purity grade available and were prepared as directed.

### 4.2. Central Composite Design 

Two-factor Central Composite Design was applied to investigate the effect of independent variables, solid lipid composition (A) and Tween 80 composition (B) on three response variables; particle size (R_1_), zeta potential (R_2_) and polydispersity index (R_3_). A total of thirteen experiments were derived from CCD through Design Expert software (version 6.0, Stat ease Inc, Minneapolis, MN, USA). The experimental design included 5 replicates of the center points, 4 axial and 4 factorial points and was carried out in randomized order. The center point was repeated five times to determine the repeatability of the employed method. The data were analyzed by a response surface regression procedure. A polynomial model was chosen based on the significant terms (*p* < 0.05), the least significant lack of fit, coefficient of variance and multiple correlation coefficients provided by Design Expert software. The upper and lower limits of the independent variables are exhibited in Table 6.

#### 4.2.1. Statistical Analysis

The optimum concentration of the independent variables (lipid and surfactant) for SLN formulation was chosen based on the condition of the responses in obtaining minimum particle size, minimum polydispersity index and maximum zeta potential. The response surface behaviour was investigated for the response function (*y*) using the polynomial Equation (1) and the generalized response surface model as shown;

*Y_i_* = *β*_0_ + *β*_1_*x*_1_ + *β*_2_*x*_2_ + *β*_11_*x*_1_^2^ + *β*_22_*x*_2_^2^ + *β*_12_*x*_1_*x*_2_ (1)

where *y* is the predicted response; *β*_0_ is constant; *β*_1_ and *β*_2_ are the linear, quadratic and interaction coefficients, respectively.

The analysis of variance (ANOVA) was used to determine the significance of the differences between the independent variables. All significant independent variables effects (*p* < 0.05) were included in the reduced model. To visualize the interaction effect of the variables on the responses, three-dimensional response surface plots were composed. The R^2^ should be at least 0.8 for models with a good fit.

#### 4.2.2. Verification of the Models

A quantitative comparison using Student’s *t*-test was carried out between the theoretical prediction and the actual obtained experimental values to validate and establish the models. A *p* < 0.05 was considered significant.

### 4.3. Preparation of Solid Lipid Nanoparticles 

Hot-high-shear-homogenization with ultrasonication method was chosen for preparation of SLN to produce nano-sized particles. The lipid, Compritol 888 ATO, was melted at 75 °C (10 °C above the lipid’s melting points) prior to dispersion into the aqueous phase containing deionized water and Tween 80. Compritol 888 ATO was also pre-heated at the same temperature. For acyclovir-loaded SLN, 10 mg acycloguanosine was added into the molten lipid during preparation and the end weight of the formulation was 20 g. The mixture was pre-emulsified by using a high-shear homogenizer (Ultra-Turrax T25, IKA, Japan) set at 20,000 rpm for 5 min, followed by 10 min of 80% intensity ultrasonication. Finally, the formulations were left to cool and kept in a 25 °C chamber before further characterization. The optimized composition of the SLN formulations was determined based on the RSM regression procedure and statistical analysis provided by the Design Expert software. 

### 4.4. Size, Zeta Potential and Polydispersity Index Analysis

Particles size, zeta potential and polydispersity index measurement of the formulations were carried out by using a dynamic light scattering method by a particle size analyzer (Malvern Nano ZS90, Malvern, Worcs, UK). Prior to the analysis, all samples were diluted (1:100) with deionized water. The diluted samples were placed in disposable cuvettes for size and PdI measurement or injected into a folded capillary electrophoresis cell for zeta potential measurement. DLS data were generated at 25 °C with a fixed 90° light incidence angle. All measurements for SLN samples were replicated three times. 

### 4.5. Drug Entrapment Efficiency

Drug entrapment efficiency (EE%) was determined by separating the free drug from SLN using the ultrafiltration/centrifugation technique. Samples were diluted in distilled water (1:200) and transferred into centrifugal filter devices. Centrifugation was performed using a multifunction centrifuge with a fixed 23° angle rotor and 5000 rpm for 10 min. The unentrapped acyclovir presented in the supernatant stored in the centrifuge tube was quantified using a UV spectrophotometer at 254 nm. %EE were calculated using the following Equation (2):

(2) $$\mathrm{EE}\%=\frac{\mathrm{Total}\;\mathrm{amount}\;\mathrm{of}\;\mathrm{drug}\;-\;\mathrm{unentrapped}\;\mathrm{drug}}{\mathrm{Total}\;\mathrm{amount}\;\mathrm{of}\;\mathrm{drug}}\times 100$$

where the total amount of drug is the amount of drug added into the lipid phase, and the unentrapped drug is the amount of drug present in the aqueous phase of the formulation measured after samples were centrifuged and filtrated.

### 4.6. Transmission Electron Microscopy

All samples were visualized using a transmission electron microscope (H-7100 Hitachi Ltd., Tokyo, Japan). Prior to TEM observation, samples were diluted 1:100 in deionized water and a drop of the sample was deposited on a 3 mm carbon-coated copper grid. The samples were negatively stained with 2% (*w*/*v*) uranyl acetate solution for 2 min, and excess staining liquid was drained off using filter paper. Samples were left to dry at room temperature prior to observation, and images were captured using the built-in camera system.

### 4.7. Differential Scanning Calorimetry

Thermal analysis was carried out using a differential scanning calorimeter (DSC), DSC822e instrument (Mettler Toledo, Zurich Switzerland). Helium was used as the purge gas, supplied at a rate of 50 mL/min and an empty aluminum pan was used as a reference. The melting point, melting enthalpy and onset temperature of an endothermic drop of the samples were calculated by the software provided by Mettler Toledo. The following Equation (3) was employed to calculate the recrystallization index (*RI*) or degree of crystallinity for all formulations:

(3) $$RI\left(\%\right)=\frac{\Delta H\;\mathrm{SLNs}\;\left(J/g\right)}{\Delta H\;\mathrm{bulk}\;\mathrm{material}\;\left(J/g\right)\;\times \;\mathrm{Concentration}\;\mathrm{lipid}\;\mathrm{phase}\;\left(\%\right)}\times \;100$$

where ∆H SLN is the melting enthalpy of Compritol 888 ATO dispersion and ∆H bulk material is the melting enthalpy of bulk lipid.

### 4.8. In Vitro Release Study

The in vitro release study for acyclovir-loaded SLN was performed in simulated gastrointestinal fluid (GIF, pH 1.2) and intestinal fluid (SIF, pH 6.8) without enzyme, prepared according to United States Pharmacopeia. The optimized acyclovir-loaded SLN or commercial suspension (equivalent to 5 mg of acyclovir) were filled into the dialysis bag (cellulose membrane, molecular weight cut-off of 12,000 Da, Sigma Aldrich, St. Louis, MO, USA) and immersed into 50 mL of release media, magnetically stirred at 100 rpm and maintained at body temperature of 37 ± 0.5 °C throughout the experiment. At selected time intervals (0.5, 1.0, 1.5, 2.5, 3.0, 4.0, 5.0, 6.0, 8.0, 10.0, 12.0 and 24.0 h), 5 mL aliquots of release media was withdrawn from the beaker. Each time after sample collection, an equal amount of fresh release media was refilled to maintain the sink condition. The amount of acyclovir released from the dialysis bag was determined using a UV spectrophotometer with UV absorbance set at 254 nm (UV-1800 UV-VIS Shimadzu, Kyoto, Japan). The in vitro drug release for all formulations was conducted in triplicate (*n* = 3), and the release profile of acyclovir from SLN was compared with commercial suspension.

### 4.9. Short-Term Stability Test

In order to assess the stability of the drug-free (blank) and acyclovir-loaded SLN dispersions, all SLN samples were stored in an amber-colored bottle for 90 days at three different temperatures; 4 ± 2 °C (refrigerated temperature), 25 ± 2 °C (chamber at room temperature) with 60 ± 5% relative humidity and 40 ± 2 °C with 75 ± 5% relative humidity (chamber) following the method described in previous studies with slight modification [54,55]. The particle size, polydispersity index (PdI) and zeta potential were measured and analyzed periodically (monthly). All measurements were repeated in triplicate.

### 4.10. Animal

A total of 24 male Sprague Dawley rats weighing approximately 200–250 g were purchased from Takrif Bistari Enterprise (Seri Kembangan, Selangor, Malaysia). Rats were housed individually in a standard laboratory polypropylene/polycarbonate rat cage with wood shaving sawdust bedding materials (Living World, Malaysia). All animals were kept on a 12 h light/dark cycle with standard laboratory conditions (controlled temperature of 24 ± 2 °C and 60 ± 5% relative humidity). Animals had free access to commercial rat pellets (Gold Coin, Malaysia) and water at all times. All procedures of the experiment had been approved by the Institutional Animal Care and Use (IACUC) of the University Putra Malaysia (UPM, Malaysia) and were carried out by following the Universiti Putra Malaysia code of practice for the care and use of animals for scientific purposes. The approval number for animal ethics is UPM/IACUC/AUP-R047/2014. Animals were randomly assigned into four groups (*n* = 6) and administered using an oral feeding (gavage) with either (1) saline (control); (2) drug-free SLN; (3) acyclovir-loaded SLN (equivalent to 20 mg/kg of acyclovir) or (4) 20 mg/kg of commercial acyclovir suspension. 

### 4.11. Blood Sample Collection and Plasma Preparation

All blood samples were withdrawn from the tail vein at 0 (pre-treatment), 0.5, 1.0, 2.5, 4.0, 6.0, 10.0 and 24.0 h post administration of the designated formulation and collected in a heparinized microcentrifuge tube. The samples were then centrifuged at 2000 g for 15 min, and the plasma samples were kept at −20 °C freezer until further analysis.

### 4.12. Ultra-Performance Liquid Chromatography (UPLC)

An Acquity ultra-performance liquid chromatographic system (Waters, Milford, MA, USA) was used to measure the estimated concentration of acyclovir in the plasma samples following the method in previous studies [56,57]. The UPLC was equipped with a photodiode array (PDA) detector and a quaternary solvent delivery system. Data were processed using the chromatographic software, Empower 3 (Waters, Milford, MA, USA). UPLC analysis was carried out at room temperature (25 °C) using an Acquity BEH C18 (100 × 2.1 mm, 1.7 µm) column (Waters, Milford, MA, USA) with UV detection set at 254 nm, and the mobile phase flow rate was maintained at 0.20 mL/min. The mobile phase consisted of 0.02 M potassium dihydrogen phosphate and acetonitrile (97:3 *v*/*v*) with final pH of 2.5. The volume of injection was 10 μL with a total run time of four minutes for each plasma.

### 4.13. Plasma Protein Precipitation for Determination of Acyclovir Concentration

Upon UPLC analysis, frozen plasma samples were thawed at room temperature for ten minutes. Each plasma sample was mixed with 5% perchloric acid at a ratio of 1:1 for protein precipitation. Subsequently, the mixture of plasma and perchloric acid was vortexed for 30 s followed by centrifugation at 4 °C for 10 min at 10,000 rpm to allow protein sedimentation. A 0.45 µm nylon syringe filter was used to filter the collected supernatant upon injection into the UPLC system.

### 4.14. Pharmacokinetic Parameters

The time to reach maximum concentration (t_max_) and maximum drug concentration observed in plasma (C_max_) were marked from the plasma acyclovir concentration versus time plot. The half-life (t_1/2_) and elimination constant (K_e_) were calculated from the elimination phase of the graph. The area under the curve calculated up to 24 h (AUC_0–24_) and the area under the curve calculated to infinity (AUC_0–∞_) was also determined. The AUC for each sample was calculated using a linear-log trapezoidal method. The formula (4) for calculating the relative bioavailability of acyclovir in the plasma samples administered via the oral route are shown below,

(4) $$\mathrm{relative}\;\mathrm{bioavailability}\;=\;\frac{{\left(\mathrm{AUC}\right)}_{A}\times {\mathrm{dose}}_{B}}{{\left(\mathrm{AUC}\right)}_{B}\times {\mathrm{dose}}_{A}}\;\times 100$$

where (AUC)_A_ is the area under the curve of the test formulation and (AUC)_B_ is the area under the curve of the reference formulation.

### 4.15. Collection of Organ Samples

At the end of in vivo experiments, all animals were sacrificed. A single lobe (median lobe) of the liver and both kidneys of the rats were excized for microscopic and histopathological analysis to observe any substantial structural changes in the organs as a result of the oral acyclovir formulations treatment. All organs were fixed and incubated in 10% formalin for more than 72 h upon the tissue processing procedure to preserve the cells and tissue components. 

### 4.16. Tissue Processing

Following fixation, a small sample (1 cm^3^) of tissues from rats in each formulation group was sliced and processed in an automatic Leica Tissue Processor ASP300 S (Wetzlar, Germany). The tissue processor was programmed to run regularly. Subsequently, using a Microm H330 microtome (Walldorf, Germany), all tissue samples were embedded in paraffin wax blocks and sectioned at 4 µm. For histological staining, the thin tissue sections were fixed on glass slides. All sections were stained with hematoxylin and eosin (H&E) using an automated tissue stainer XL (Leica, Wetzlar, Germany) prior to light microscopic examination, as described in previous work [58].

### 4.17. Light Microscopy

Histological observation and evaluation of the tissue morphology (any physical changes to the kidney and liver tissues due to administration of various acyclovir formulations) were carried out using an Olympus BX40 light microscope fitted with an Olympus DP 70 digital camera (Olympus Corporation, Tokyo, Japan). The stained sections were examined under 20× and 40× magnifications. All selected images of the tissues were captured using a digital image analyzer, Olympus Soft Imaging Solution Cell^F software (Olympus Corporation, Tokyo, Japan).

## 5. Conclusions

The optimization process of the SLN was successfully carried out to produce a suitable nano-sized drug carrier for oral delivery of acyclovir. Data of the study suggest that the optimized formulations of Compritol 888 ATO SLN using the CCD approach were appropriate and valid. The relationship between surfactant and solid lipid as the main composition of SLN formulation and their impact on the size, zeta potential and PdI were well understood from the response surface plots generated in this study. Therefore, the optimized compositions of SLN dispersion as suggested by RSM were satisfactory. The evaluation on the percentage of entrapment efficiency showed relatively good entrapment of acyclovir, and the formulation was stable for at least up to 3 months at refrigerated and room temperature. It could be concluded from this in vivo pharmacokinetic study that the acyclovir-loaded SLN was proven to prolong the release of acyclovir and enhance the relative oral bioavailability of acyclovir in plasma.

## Figures and Tables

**Table 1:** Equations, regression coefficients and probability values for the final reduced model.

Size, R_1_	Equation: 93.92 + 44.30*A* − 5.93*B* + 14.38*A*^2^ + 51.75*B*^2^ − 36.38*AB* *R*^2^ value: 0.9997 *p*-value: <0.0001
Zeta Potential, R_2_	Equation: −40.20 + 2.24*A* − 1.25*B* − 0.36*A*^2^ − 1.53*B*^2^ − 1.25*AB* *R*^2^ value: 0.9620 *p*-value: 0.0029
Polydispersity Index, R_3_	Equation: 0.20 − 0.04*A* − 0.07*B* + 0.06*A*^2^ + 0.07*B*^2^ − 0.048*AB* *R*^2^ value: 0.9983 *p*-value: <0.0001

A: Compritol 888 ATO composition; B: Tween 80 composition.

**Table 2:** ANOVA of regression coefficient of the fitted quadratic equation for SLN.

Variables		Size		Zeta Potential		PdI	
		F Value	*p*-Value	F Value	*p*-Value	F Value	*p*-Value
Main Effects	A	473.19	<0.0001	6.65	0.0495	27.73	0.0033
	B	8.48	0.0333	2.08	0.2084	430.11	<0.0001
Quadratic Effects	A^2^	433.32	<0.0001	1.47	0.2801	540.82	<0.0001
	B^2^	5614.36	<0.0001	27.08	0.0035	735.67	<0.0001
Interaction Effect	AB	1594.82	<0.0001	10.38	0.0234	194.09	<0.0001

A: Compritol 888 ATO composition; B: Tween 80 composition.

**Table 3:** Predicted and observed response value for the optimized SLN.

Responses	Predicted	Observed
Particle Size (nm)	100.00	104.89
Polydispersity Index	0.22	0.21
Zeta Potential (mV)	−40.01	−37.00

**Table 4:** Size, PdI and zeta potential measurement of SLN formulations stored at 4, 25 and 40 °C.

	Drug-Free SLN				Acyclovir-Loaded SLN			
	Freshly Prepared	1 Month	2 Month	3 Month	Freshly Prepared	1 Month	2 Month	3 Month
**Size (nm)**								
4 °C	104.89 ± 5.53	104.11 ± 5.90	106.85 ± 3.90	106.95 ± 4.42	108.68 ± 1.03	105.05 ± 0.72	106.78 ± 1.79	108.33 ± 1.28
25 °C		102.28 ± 3.42	103.33 ± 2.63	102.68 ± 1.04		106.85 ± 1.53	111.38 ± 3.76	113.05 ± 1.79
40 °C		255.70 ± 8.73	292.62 ±14.68	330.55 ± 9.73		128.43 ± 5.19	141.43 ±10.53	622.98 ±17.17
**PdI**								
4 °C	0.21 ± 0.01	0.22 ± 0.03	0.22 ± 0.01	0.21 ± 0.01	0.22 ± 0.03	0.20 ± 0.02	0.21 ± 0.02	0.21 ± 0.01
25 °C		0.20 ± 0.01	0.20 ± 0.01	0.20 ± 0.02		0.20 ± 0.01	0.20 ± 0.02	0.21 ± 0.01
40 °C		0.33 ± 0.02	0.43 ± 0.02	0.46 ± 0.01		0.29 ± 0.02	0.37 ± 0.04	0.35 ± 0.02
**Zeta Potential** (**mV**)								
4 °C	−37.00 ± 0.89	−38.13 ± 0.85	−37.88 ± 1.36	−35.13 ± 2.21	−33.45 ± 0.78	−32.88 ± 1.01	−34.60 ± 1.28	−33.98 ± 0.87
25 °C		−35.28 ± 0.94	−35.23 ± 1.07	−36.05 ± 1.54		−33.45 ± 0.93	−33.50 ± 1.41	−34.93 ± 1.31
40 °C		−25.50 ± 0.81	−27.08 ± 1.19	−25.23 ± 1.04		−37.00 ± 1.01	−25.48 ± 1.56	−26.38 ± 0.76

**Table 5:** Pharmacokinetic parameters after oral administration of acyclovir-loaded SLN or commercial suspension.

Parameters	Commercial Acyclovir Suspension	Acyclovir-Loaded SLN
C_max_ (ng/mL)	303.50 ± 26.70	818.67 ± 66.02
T_max_ (h)	1.00 ± 0.00	1.00 ± 0.00
AUC_0–24_ (h.ng.mL^−1^)	1243.75 ± 125.90	5759.00 ± 346.40
AUC_0–∞_ (h.ng.mL^−1^)	1341.67 ± 133.40	6783.14 ± 313.80
K_e_ (h^−1^)	0.37 ± 0.05	0.15 ± 0.02
t_1/2_ (h)	2.06 ± 0.29	5.53 ± 0.99

**Table 6:** Independent variables with high and low levels.

Independent Variables	Coded Levels				
	Axial (−α)	Low	Centre	High	Axial (+α)
Compritol 888 ATO (mg)	117.16	200.00	400.00	600.00	682.84
Tween 80 (% *w*/*w*)	0.17	1.00	3.00	5.00	5.83

## Data Availability

The data presented in this study are available on request from the corresponding author.

## Supplementary Materials

The following are available online, Figure S1: Overview of experiment workflow.

## Sample Availability

Samples of the formulations are no longer available from the authors due to limited shelf-life.
