# Supplementary material for: Central Composite Design for Formulation and Optimization of Solid Lipid Nanoparticles to Enhance Oral Bioavailability of Acyclovir
Source: Molecules. 2021 Sep 7;26(18):5432. doi: 10.3390/molecules26185432 (PMC8470285; doi:10.3390/molecules26185432)
Supplement: Supplementary file 1 [file molecules-26-05432-s001.zip › molecules-1354305-supplementary.pdf]

molecules

MDPI

Supplementary Materials

Article

Central Composite Design for Formulation and Optimization of Solid Lipid Nanoparticles to Enhance Oral Bioavailability of Acyclovir

Haniza Hassan [1,*], Siti Khadijah Adam [1], Ekram Alias [2], Meor Mohd Redzuan Meor Mohd Affandi [3], Ahmad Fuad Shamsuddin [4] and Rusliza Basir [1]

[1] Department of Human Anatomy, Faculty of Medicine and Health Sciences, University Putra Malaysia (UPM), Serdang 43400, Selangor, Malaysia; sk.adam@upm.edu.my (S.K.A.); rusliza@upm.edu.my (R.B.)
[2] UKM Medical Centre, Department of Biochemistry, Faculty of Medicine, Universiti Kebangsaan Malaysia, Jalan Yaakob Latiff, Bandar Tun Razak 56000, Kuala Lumpur, Malaysia; ekram.alias@ppukm.ukm.edu.my
[3] School of Pharmacy, Puncak Alam Campus, Universiti Teknologi MARA (UiTM), Bandar Puncak Alam 42300, Selangor, Malaysia; meor@uitm.edu.my
[4] Faculty of Pharmacy and Health Sciences, Universiti Kuala Lumpur Royal College of Medicine Perak, 30450 Ipoh, Perak, Malaysia; fuad.shamsuddin@unikl.edu.my
* Correspondence: nizahassan@upm.edu.my Tel.: +60-3-97692665

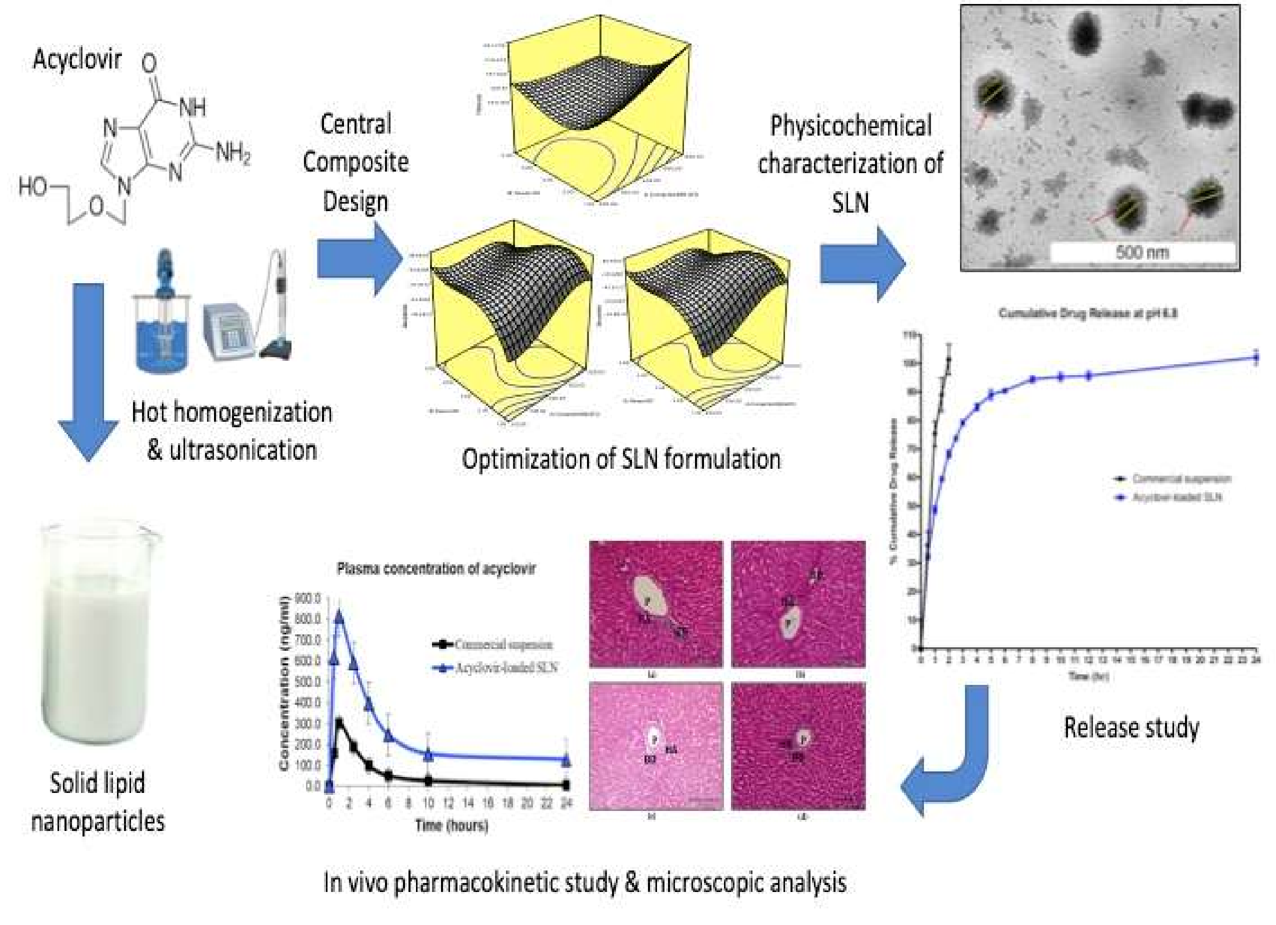

Figure S1. Overview of experiment workflow.
